# Combining adoptive NK cell infusion with a dopamine-releasing peptide reduces senescent cells in aged mice

**DOI:** 10.1038/s41419-022-04562-w

**Published:** 2022-04-05

**Authors:** Zongke Bai, Peiwei Yang, Fan Yu, Zhong Li, Zheng Yao, Jean Martinez, Mengwei Li, Hanmei Xu

**Affiliations:** 1 The Engineering Research Center of Synthetic Polypeptide Drug Discovery and Evaluation, Jiangsu Province, China Pharmaceutical University, Nanjing, 210009 P.R. China; 2 State Key Laboratory of Natural Medicines, Ministry of Education, China Pharmaceutical University, Nanjing, 210009 P.R. China; 3 Shanghai Engineering Research Center for Cell Therapy, Shanghai, 201805 China; 4 Faculté de Pharmacie, Institut des Biomolécules Max Mousseron (IBMM) UMR 5247, Université de Montpellier, CNRS, ENSCM, Montpellier, France

**Keywords:** Immunotherapy, Diseases

## Abstract

Aging inducing the development of senescent cells (SNCs) in various tissues is considered as the main cause of the age-related diseases. Senotherapy has become a promising anti-aging therapy. However, the effectivity and side-effect of senolytic agents are still concern. Here, we observed the downregulation of senescence-related genes by adoptive infusion of natural killer (NK) cells in 26 cases in peripheral blood CD3^+^ T cells. NK cell treatment also significantly decreased levels of senescence markers and senescence-associated secretory phenotypes (SASPs) in three senescent adipose tissues when culturing them together. Interestingly, cytotoxic activity of mouse NK cells against SNCs was significantly enhanced by dopamine in vitro through D1-like receptors. Acein, dopamine-releasing peptide, promoted the adoptive infusion of NK cells in effectively eliminating SNCs in a variety of tissues and reduced local and systemic SASPs in aging mice but Acein alone did not have the senolytic effect. These data demonstrated that adoptive infusion of NK cells is an effective means in removing SNCs, and peptide Acein combined with NK cells further enhances this effect in aging mice.

## Introduction

Aging is one of the major risk factors for many diseases [1], and the development of methods to intervene in aging will significantly reduce the risk of a series of age-related diseases such as cardiovascular and cerebrovascular diseases [2, 3], cancer [4], and Alzheimer’s disease [5]. Senescent cells (SNCs) gradually accumulate in tissues during the aging process and are considered the main cause of age-related diseases [6–8]. The removal of SNCs in mouse models has been shown to prevent or delay tissue dysfunction and extend healthy lifespans [9, 10]. In contrast, transplanting small amounts of SNCs causes physiological dysfunction in young mice [11]. These studies have opened doors for the development of methods that target the removal of SNCs to ameliorate age-related chronic diseases.

Targeting clearance of SNCs, termed “senolytics” was the first senotherapy successfully tested in a preclinical in vivo model, and several senolytic agents currently exist [12]. Many of these drugs target up regulated anti-apoptotic pathways in SNCs to selectively clear certain types of SNCs, and have shown the potential to extend lifespan and improve body functions [13, 14]. Despite the successful reversal of age-related pathology in animal models, there may be some obstacles of using senolytic drugs in humans due to the heterogeneity of SNCs and the high risk of toxicity [15]. Therefore, an alternative method that can safely eliminate a wide range of SNCs in humans should be explored.

SNCs can be recognized and removed by the immune system [16]. Previous studies have shown that SNCs activate natural killer (NK) cells by up regulating the major histocompability class I chain-related protein A and B activating ligand [17]. However, with increasing age, the efficiency of the immune system decreases, which can lead to the immune escape of SNCs [18]. Methods to overcome immune escape caused by decreased immune function have been explored in cancer therapy [19]. Recent progress has been made in adoptively transferring NK cells to eliminate tumours, which has shown some efficacy [20]; thus, it was reasonable to assume that the adoptive infusion of NK cells might produce cytotoxicity in SNCs.

The nervous and immune systems are the two most important adaptive systems of the body. Several studies have shown that dopamine (DA) as an immune regulator is a key to the neuroimmune communication [21]. DA performs its biological functions by interaction with and activation of dopamine receptors (DR), which are divided into 2 subgroups, D1-like (D1 and D5), and D2-like (D2, D3 and D4). In terms of their different functions, the engagement of D1-like DR stimulates cAMP production, while the engagement of D2-like DR inhibits cAMP production [22]. Previous studies have shown that D1-like DR stimulation enhances the cytotoxicity of NK cells both in vitro [23] and in vivo [24]. However, DA levels drop as human age increase [25]. Thus, we hypothesized that dopaminergic drugs could enhance cytotoxicity of the adoptive infusion of NK cells.

Here, we propose the use of the nonapeptide Acein, which interacted with angiotensin converting enzyme (ACE I) to induce DA secretion [26], in combination with systemic NK cell therapy to eliminate SNCs. In vitro results showed that NK cells removed SNCs, independently of senescence inducers and cell types. In an aging mouse model, NK cell therapy in combination with Acein significantly reduced the number of senescence-associated β-galactosidase (SA-β-gal)-positive cells in multiple tissues, decreased the expression of senescence-associated genes in major organs, and alleviated senescence-associated secretory phenotypes (SASPs). The results of this study provide insights into possible restoration of the immune surveillance of chronic SNCs using NK cell therapy in combination with Acein.

## Results

### Adoptive infusion of NK cells reduces senescent CD3^+^ T cells in peripheral blood

**Fig. 1:**
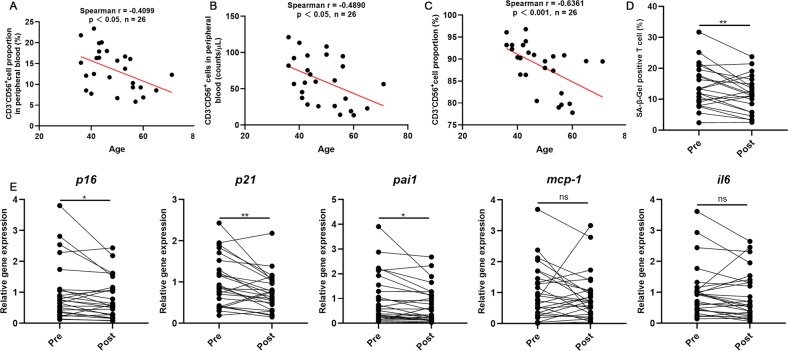
Adoptive infusion of NK cells reduces senescence in peripheral blood T cells. **A** Correlation analyses between NK cell proportion and age (*n* = 26). **B** Correlation analyses between NK cell numbers and age (*n* = 26). **C** Correlation analyses between purity of the NK cell product reinfused into each individual and age (*n* = 26). The spearman correlation analysis was used in all cases. The correlation was significant at 0.05 (two-sided). **D** CD3^+^ T cells were separated and SA-β-gal staining was performed pre- and post-infusion of NK cells (*n* = 26). **E** Expression of senescence (P16^Ink4a^ and P21) (n = 25) and SASP markers (IL-6, monocyte chemoattractant protein-1 [MCP-1], plasminogen activator 1 [Pai1]) (*n* = 26) in CD3^+^ T cells were analysed by qRT-PCR. Differences were assessed by the two-tailed paired Student’s *t*-test. ns *P* > 0.05, *P < 0.05, **P < 0.01. Two parallel samples were set in each experiment and three independent experiments were performed for each result.

Twenty-six volunteers who received NK cell infusion were recruited for the study. The characteristics and NK cell properties of the volunteers are presented in Table 1. The time interval for blood recollection after NK cell transfusion was about 37 (25–57) days. The proportion (Fig. 1A) and absolute number (Fig. 1B) of NK cells in the peripheral blood was inversely related to the age of the volunteers. Surprisingly, the purity of the NK cell product reinfused into each individual was also inversely correlated with the age of the volunteers (Fig. 1C). This may be due to the gradually decreased number of NK cells with increasing age. In order to reflect the anti-aging effect of NK cell administration, multiple senescent markers in peripheral blood CD3^+^ T cells, which largely reflects the age-related phenotype of the body [27], were detected before and after NK cell infusion. Compared with the senescence markers and SASP factor of CD3^+^ T cells in peripheral blood before and after autologous NK cell infusion, the proportion of SA-β-gal-positive T cells was significantly decreased after infusion (Fig. 1D). The mRNA levels of P16, P21, and plasminogen activator inhibitor 1 (Pai1) were significantly decreased, while the changes in mRNA levels of monocyte chemoattractant protein-1 (mcp-1) and IL-6 were not significant (Fig. 1E). There was no significant difference in the biochemical indexes (Supplementary Table 1) in the peripheral blood.

**Table 1:** Clinical characteristics of 26 individuals receiving NK cells administration.

Characteristics	
Mean age (range)	46.5 (36–71)
Gender	
Male	10
Female	16
Cell counts (range, ×10^9^)	4.15 (3.76–5.87)
Phenotype (% CD3–CD56^+^)	89.52 (77.81–96.81)
Cell viability (%)	95.1 (87.8–98.6)
Sterility test	Negative (26/26)
Mycoplasma detection	Negative (26/26)
Endotoxin detection	<0.5 EU/mL (26/26)

### Human NK cells reduce adipose tissue senescence markers and secretion of proinflammatory cytokines

**Fig. 2:**
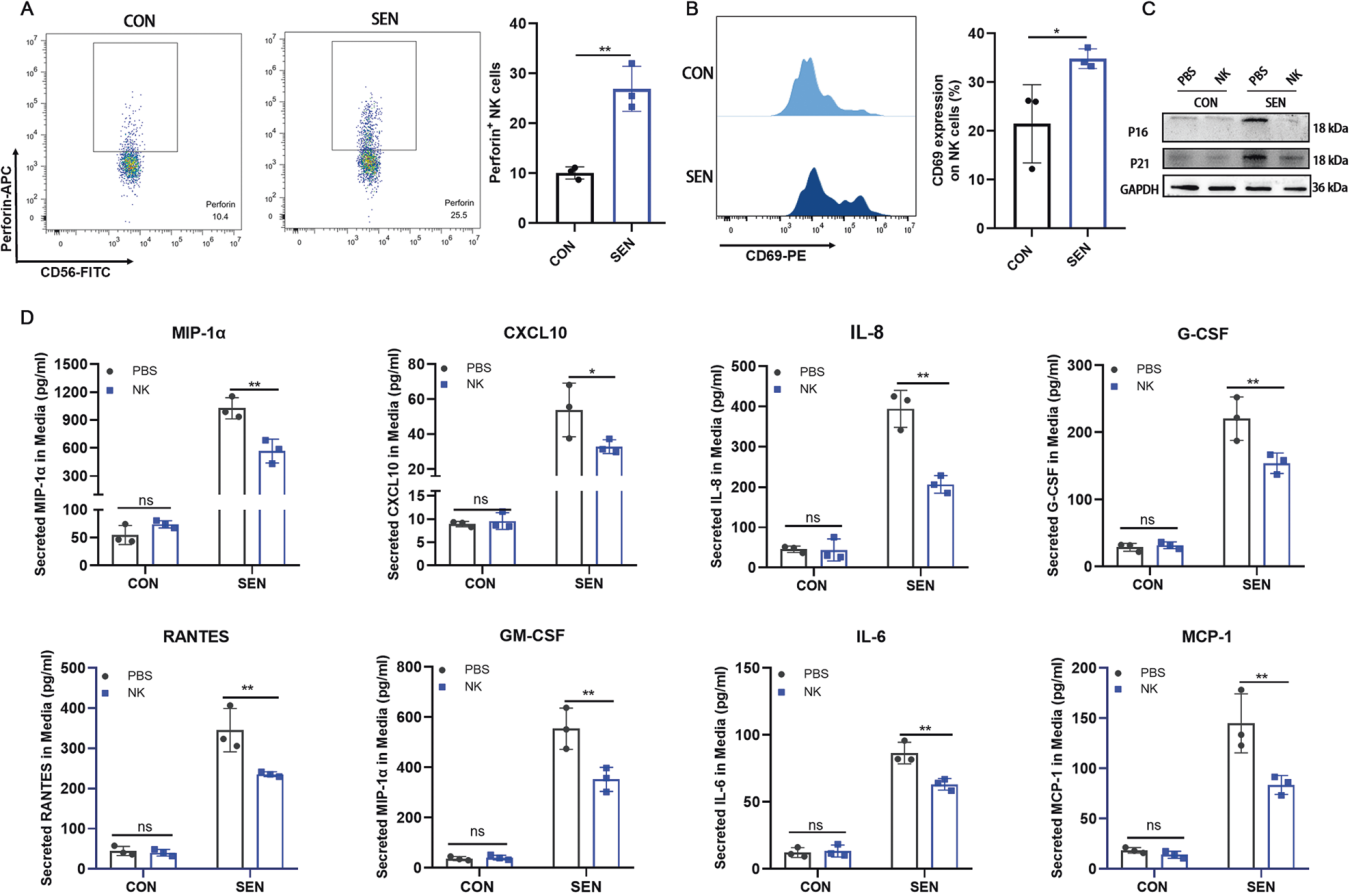
NK cells reduce senescent markers and decrease the SASP phenotype in human adipose tissue. Flow cytometry to detect expression of (**A**) perforin and (**B**) CD69 was performed after 48-h co-incubation with NK cells and human adipose tissue, treated with conditional medium from control (CON) or senescent (SEN) preadipocytes for 24 h. Cells were gated in CD3^-^CD56^+^ location. n = 3. Data are presented as means ± SD. Differences were assessed by the two-tailed unpaired nonparametric *t*-test. *P < 0.05, **P < 0.01. **C** Senescent markers for P16 and P21 were detected by Western blotting. *n* = 3. **D** Secreted proinflammatory cytokines in conditional medium from adipose tissue was collected 48-h after removing the NK cells. *n* = 3. Data are presented as means ± SD. Differences were assessed by the two-way ANOVA test. ns *P* > 0.05, *P < 0.05, **P < 0.01. Two parallel samples were set in each experiment and three independent experiments were performed for each result.

Adipose tissue was collected from three obese individuals, as obesity tends to cause accumulation of SNCs [28]. In addition, the adipose tissue was cultured in conditioned medium from SNCs to further induce adipose tissue senescence. Adipose tissue cultured in conditioned medium from non-SNCs was considered as the control. Perforin (Fig. 2A) and CD69 (Fig. 2B) expression in NK cells was significantly up regulated after co-incubation with senescent adipose tissue compared to co-incubation with control adipose tissue. The expression of senescent markers p16 and p21 in senescent adipose tissue was significantly decreased after NK cell treatment (Fig. 2C, Supplementary Fig. 1A, B). We also found that the key component of SASP in the conditioned medium after co-incubation with NK cells in the senescent group was significantly decreased (Fig. 2D). These results suggested that NK cells could eliminate SNCs from adipose tissue and reduce the SASP phenotype.

### Mouse NK cells can be activated against SNCs and exert cytotoxicity

**Fig. 3:**
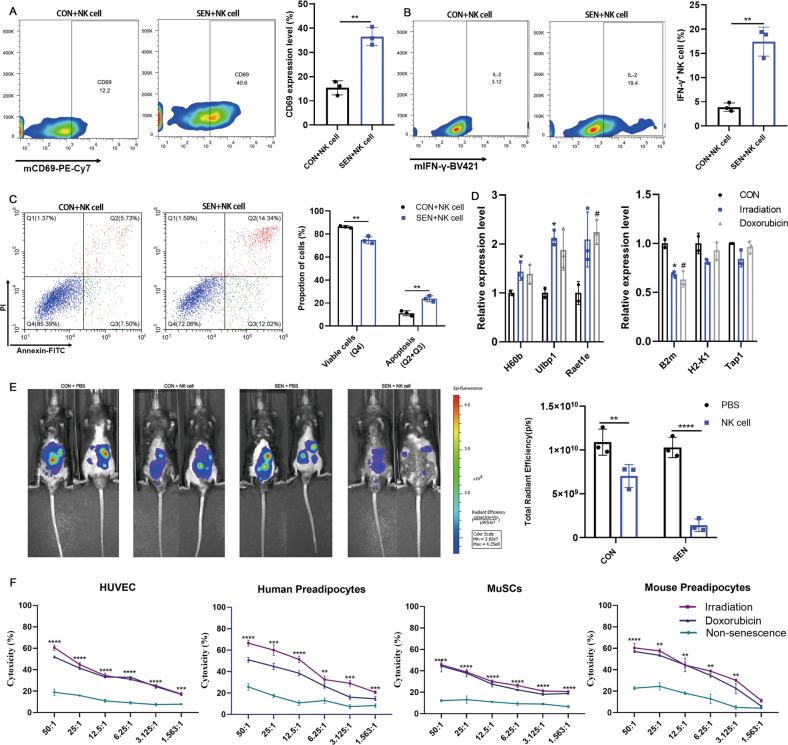
SNCs activate NK cells and were killed by NK cells. Flow cytometry to detect the expression of (**A**) CD69 and (**B**) IFN-γ was performed after a 24 h co-incubation with mouse NK cells and preadipocytes or irradiation-induced preadipocytes. *n* = 3. Data are presented as means ± SD. Differences were assessed by the two-tailed unpaired nonparametric *t*-test. **P < 0.01. **C** SNC apoptosis was detected after a 24-h co-incubation with DiR-labeled NK cells by flow cytometry. *n* = 3. Data are presented as means ± SD. Differences were assessed by the two-way ANOVA test. **P < 0.01. **D** Activating ligands and inhibitory ligands of NK cells on preadipocytes, doxorubicin-induced preadipocytes or irradiation-induced preadipocytes were measured by qPCR. *n* = 3. Data are presented as means ± SD. Differences were assessed by the one-way ANOVA test. *P < 0.05 (Irradiation vs CON). #*P* < 0.05 (Doxorubicin vs CON). **E** 1 × 10^6^ DiR-labeled control preadipocytes or irradiation-induced senescent preadipocytes were implanted into the abdominal cavity. Representative images showing fluorescence activity in mice seven days after 5 × 10^6^ NK cell treatment. Quantification of fluorescence activity. *n* = 3. Data are presented as means ± SD. Differences were assessed by the two-way ANOVA test. **P < 0.01, ****P < 0.0001. **F** Quantification of cytotoxicity of non-senescent and senescent counterparts induced by irradiation or adriamycin incubated with increasing NK cells for 24 h. *n* = 3. Data are presented as means ± SD. Differences were assessed by the two-way ANOVA test. **P < 0.01, ***P < 0.001, ****P < 0.0001. Two parallel samples were set in each experiment and three independent experiments were performed for each result.

We first induced the senescence of mouse adipose progenitor cells by irradiation or Adriamycin as previous described [29]. To determine whether NK cells were specifically activated by SNCs, non-irradiated control cells or irradiated SNCs were co-incubated with NK cells for 24 h. Flow cytometry showed that the expression levels of CD69 (Fig. 3A) and IFN-γ (Fig. 3B) in NK cells were significantly up regulated in SNC target cells compared to control target cells. Meanwhile, after co-incubation with NK cells, the apoptotic level of SNCs was significantly higher than that of control cells (Fig. 3C). Next, we detected the expression of NK cell activating and inhibitory ligands in the SNCs. The qPCR results showed that the expression level of activating ligands in NK cells was significantly up regulated compared to control cells, and the expression level of some inhibitory ligands, such as beta 2 microglobulin (β2m), was down regulated compared to control cells (Fig. 3D). We have also examined the ability of NK cells to eliminate SNCs in vivo. DiR-labeled control cells and DiR-labeled SNCs were transplanted into the abdominal cavity of mice, and NK cells were administered through the tail vein. In vivo imaging results showed that the fluorescence intensity in mice transplanted with SNCs after NK cell treatment was significantly weaker than that in mice transplanted with control cells (Fig. 3E). This indicated that the ability of NK cells to kill SNCs was significantly higher than that of SNCs in vivo. Next, we determined whether the cytotoxicity of NK cells against SNCs was dose-dependent and cell-specific. At the same time, we also used doxorubicin-induced senescence to determine whether the cytotoxicity of NK cells against SNCs was limited to radiation induction. The results showed that NK cells had significant cytotoxic activity against SNCs in a dose-dependent manner, but little cytotoxicity against control cells. Different senescence stimulation methods had no effect on the ability of NK cells to kill SNCs (Fig. 3F). These results suggested that NK cells could be specifically activated by SNCs and produce significant cytotoxicity against SNCs in vitro and in vivo.

### DA enhances the cytotoxicity of mouse NK cells against SNCs through D1-like receptors

**Fig. 4:**
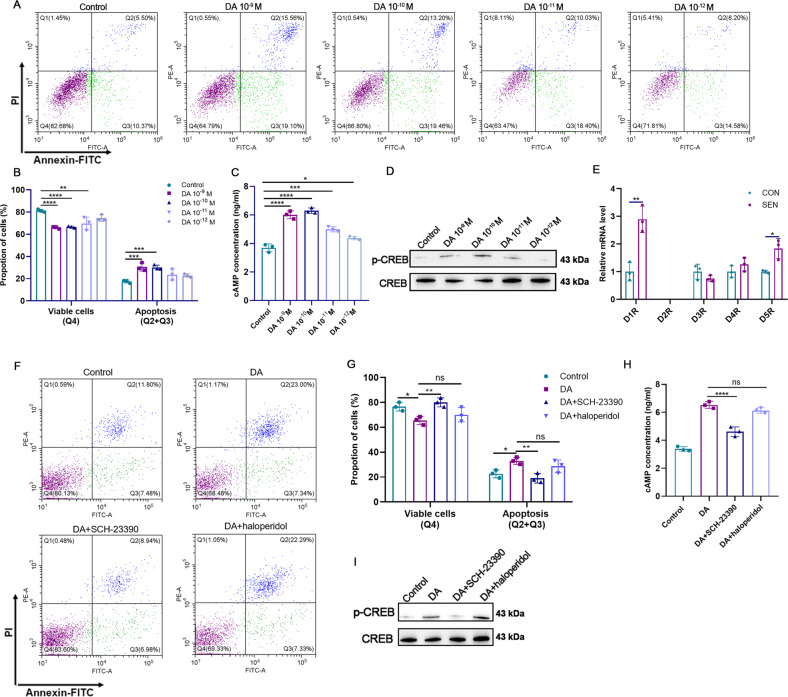
DA enhances mouse NK cell cytotoxicity against SNCs through D1-like receptors. **A**, **B** Cytotoxicity of mouse NK cells against SNCs supplemented with different concentrations of DA after a 24-h co-incubation. *n* = 3. Data are presented as means ± SD. Differences were assessed by the two-way ANOVA test. **P < 0.01, ***P < 0.001, ****P < 0.0001. **C** Data showing cAMP content in NK cells after 24-h incubation with SNCs supplemented with different concentrations of DA. *n* = 3. Data are presented as means ± SD. Differences were assessed by the one-way ANOVA test. *P < 0.05, ***P < 0.001, ****P < 0.0001. **D** Western blot analysis of phosphorylated CREB level in NK cells after 24-h incubation with SNCs supplemented with different concentrations of DA. **E** DR expression in NK cells was detected after co-incubation with SNCs or control cells by qPCR. *n* = 3. Data are presented as means ± SD. Differences were assessed by the two-tailed unpaired nonparametric *t*-test. *P < 0.05, **P < 0.01. **F**, **G** Cytotoxicity of mouse NK cells against SNCs supplemented with 10^−9^ M DA and SCH23300 or haloperidol after a 24-h co-incubation. *n* = 3. Data are presented as means ± SD. Differences were assessed by the two-way ANOVA test. ns *P* > 0.05, *P < 0.05, **P < 0.01. **H** cAMP content in NK cells after incubation with SNCs supplemented with 10^−9^ M DA and SCH23300 or haloperidol after a 24-h co-incubation. *n* = 3. Data are presented as means ± SD. Differences were assessed by the one-way ANOVA test. ns *P* > 0.05, ****P < 0.0001. **I** Western blot analysis of phosphorylated CREB level in NK cells after incubation with SNCs supplemented with 10^−9 ^ M DA and SCH23300 or haloperidol after a 24-h co-incubation. Two parallel samples were set in each experiment and three independent experiments were performed for each result.

In view of the important neuroimmune communication function of DA [30], we assessed whether DA could enhance the cytotoxicity of mouse NK cells against SNCs. NK cells were co-incubated with radiation-induced SNCs, and different concentrations of DA were added to the medium. The results showed that DA could enhance the cytotoxicity of NK cells against SNCs (Fig. 4A, B). To characterize the signalling pathway by which DA enhanced the killing effect of NK cells on SNCs, the changes in DR expression, cAMP content, and cAMP response element-binding protein (CREB) phosphorylation in NK cells were evaluated. In NK cells co-incubated with SNCs along with DA, the content of cAMP (Fig. 4C) and the phosphorylation level of CREB (Fig. 4D, Supplementary Fig. 2A) were significantly increased, compared with NK cells and SNC co-incubation without DA. In order to clarify why DA enhances the killing activity of NK cells against SNCs, we compared the changes of DR on NK cells after NK cells were co-incubated with SNCs or control cells. The results showed that the D1 and D5 DR expression level was significantly up regulated after NK cells were co-incubated with SNCs compared to control cells (Fig. 4E). Next, DA and D1-like receptor antagonists or D2-like receptor antagonists were added together in the co-incubation system of NK cells and SNCs. Compared with the addition of DA alone, the apoptosis level of SNCs was decreased upon addition of DA + SCH-23300 (D1-like receptor antagonist) (Fig. 4F, G). Meanwhile, the cAMP content (Fig. 4H) and the phosphorylation level of CREB (Fig. 4I, Supplementary Fig. 2B) in NK cells were down regulated. By contrast, the addition of DA + haloperidol (D2 receptor antagonist) did not significantly change the apoptosis level of SNCs, the cAMP content, and the CREB phosphorylation in NK cells, compared to the addition of DA alone. These results demonstrated that DA enhanced the killing activity of mouse NK cells against SNCs through D1-like DRs.

### Acein combined with mouse NK cells significantly enhances the clearance of SNCs in vivo

**Fig. 5:**
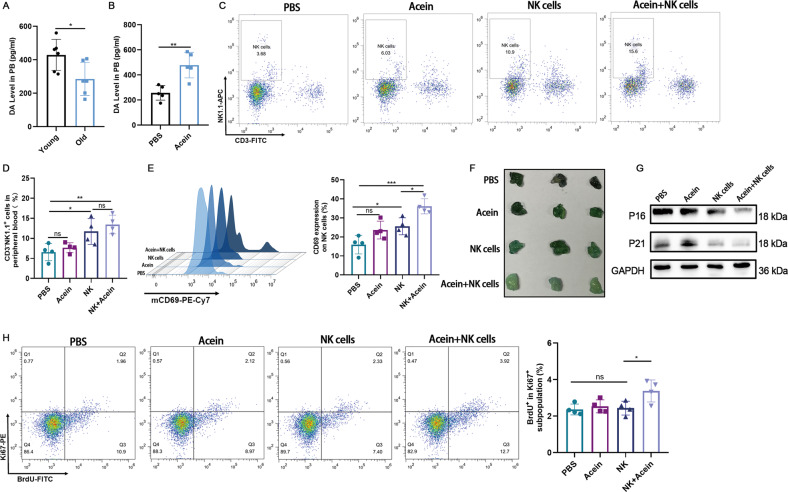
Acein enhances peripheral blood NK cell activation and reduces adipose tissue senescence. **A** Peripheral dopamine levels in young (10-week-old) and aged (20-month-old) mouse. *n* = 6. Data are presented as means ± SD. Differences were assessed by the two-tailed unpaired nonparametric *t*-test. *P < 0.05. **B** Peripheral dopamine levels induced by 10 mg/kg Acein 1 h after Acein administration in old mice. *n* = 5. Data are presented as means ± SD. Differences were assessed by the two-tailed unpaired nonparametric *t*-test. **P < 0.01. **C**, **D** NK cells in peripheral blood were detected by flow cytometry after a four-month treatment. *n* = 4. Data are presented as means ± SD. Differences were assessed by the one-way ANOVA test. ns *P* > 0.05, *P < 0.05, **P < 0.01. **E** CD69 expression level in NK cells. *n* = 4. Data are presented as means ± SD. Differences were assessed by the one-way ANOVA test. ns P>0.05, *P < 0.05, ***P < 0.001. **F** SA-*β*-gal staining of adipose tissue in the groin. **G** Expression levels of senescent markers P16 and P21 in adipose tissue in the groin. **H** BrdU was injected intraperitoneally at 24 and 72 h before euthanasia. BrdU^+^Ki67^+^adipocytes in the groin were detected by flow cytometry. *n* = 4^.^ Data are presented as means ± SD. Differences were assessed by the one-way ANOVA test. ns *P* > 0.05, *P < 0.05. Two parallel samples were set in each experiment.

Previous study has shown that dopamine decreases with age in human [22]. We first measured the peripheral dopamine level in young and old mouse. The results indicated that the plasma dopamine levels of the old mice were lower than those of the young mice (Fig. 5A). Next, peripheral dopamine release after intraperitoneal injection of Acein was explored in old mice. We found that plasma dopamine levels increased significantly after a 10 mg/kg Acein injection (Fig. 5B, Supplementary Fig. 3). In view of this, we performed of mouse NK cells combined with Acein to treat aged mice. We detected the status of NK cells in the peripheral blood of mice after treatment and found that the number of NK cells in the peripheral blood of infusion of NK cells alone or NK cells combined with Acein was significantly higher than that of the Acein treated group and PBS group, whereas the number of NK cells was not significantly different between the NK cells treated group and the NK cells combined with the Acein treated group (Fig. 5C, D). Further detection of the activation level of NK cells showed that the expression level of CD69 in peripheral blood NK cells in the combined treated group was significantly up regulated compared to the NK cells treated group (Fig. 5E). Next, we have evaluated the SNCs in the tissue. We found that infusion of NK cells alone reduced the expression of some senescent markers compared with the PBS group and Acein treated group, whereas infusion of NK cells combined with Acein significantly reduced the SA-*β*-gal-positive cells (Fig. 5F) as well as the expression levels of P16 and P21 (Fig. 5G, Supplementary Fig. 4A, B) in the adipose tissue, compared with treatment of NK cells alone. The number of Ki67^+^BrdU^+^ cells in the adipose tissue was also significantly increased (Fig. 5H) further proving that the content of SNCs in the adipose tissue was lower in the combined treated group. SA-β-gal staining of liver, lung, kidney, and fat sections also showed the lowest level of SNCs in the combined treated group (Supplementary Fig. 5A-D). These results showed that NK cells combined with Acein therapy enhanced the activation level of NK cells in mice, and caused significant elimination of SNCs in vivo.

### Acein combined with NK cells reduces age-related phenotypes in aged mice

**Fig. 6:**
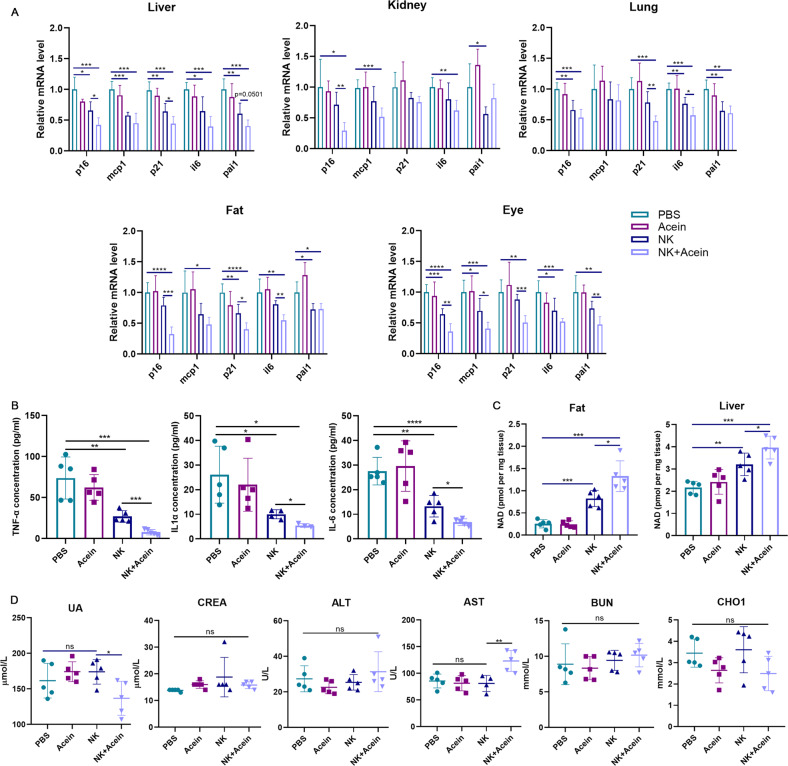
NK cells combined with Acein attenuate age-related phenotypes in aged mice. **A** Senescence (p16 and p21) and SASP markers (IL-6, MCP-1, Pai1) in different tissues detected by qRT-PCR after a four-month treatment. *n* = 5. Data are presented as means ± SD. Differences were assessed by the one-way ANOVA test. *P < 0.05, **P < 0.01. **P < 0.001, ****P < 0.0001. **B** Main SASP factor in the serum was detected by CBA after a four-month treatment. *n* = 5. Data are presented as means ± SD. Differences were assessed by the one-way ANOVA test. *P < 0.05, **P < 0.01, **P < 0.001, ****P < 0.0001. **C** NAD levels in fat and liver. *n* = 5. Data are presented as means ± SD. Differences were assessed by the one-way ANOVA test after a four-month treatment. *P < 0.05, **P < 0.01. **P < 0.001. **D** The levels of ALT, AST, CREA, UA, CHOl, and BUN in aged mice after a four-month treatment. *n* = 5. Data are presented as means ± SD. Differences were assessed by the one-way ANOVA test. ns *P* > 0.05, **P < 0.01. Two parallel samples were set in each experiment and three independent experiments were performed for each result.

To further confirm age-related phenotypes in mice, liver, lung, kidney, fat, and eye tissues were collected to detect a variety of senescent markers including P16, P21, and SASP factors. These samples were chosen because these tissues are more likely to undergo senescence with age [31]. In each tissue, after NK cell infusion alone, the mRNA levels of P16, P21 and SASP factors were significantly lower than those in the control group, whereas the levels of senescence markers in the liver, fat, and eye tissues in the combined treated group were further decreased compared to the group treated with NK cell infusion alone (Fig. 6A). Similarly, we have also detected the main SASP factor in the serum of mice, and the results were consistent with the results in tissues (Fig. 6B). In addition, we have also examined NAD levels in the liver and adipose tissue, which are linked to aging [32]. We found that infusion of NK cells alone or in combination therapy significantly increased the content of NAD in liver and adipose tissue, the level of improvement being significantly higher in the combined therapy group (Fig. 6C). Some serum biochemical indexes including ALT, AST, CREA, UA, CHOL, and BUN are associated with aging levels [33]. We have found that only UA was significantly reduced in the serum of mice in the combination treated group, while the other key biochemical indexes did not significantly differ between the treated groups (Fig. 6D). Taken altogether, these results showed that Acein combined with NK cell therapy could significantly reduce the age-related phenotypes in aging mice.

## Material and methods

### Infusion of human NK cells

Autologous NK cell infusion was performed by the Shanghai Mengchao Cancer Hospital (Shanghai, China), and all protocols were approved by their ethics committee (No.05, 2020, Medical Ethics Committee of Shanghai Mengchao Cancer Hospital). All volunteers provided signed informed consent to obtain peripheral blood. Briefly, 50 mL peripheral blood was extracted from each individual, after which peripheral blood mononuclear cells (PBMCs) were isolated and transferred to a T75 flask with ExCellerate™ Human NK Cell Expansion Media (R&D Systems, USA) containing 1×Cloudz CD2/NKp46, recombinant Human IL-2 (27 ng/mL), recombinant Human IL-12 (10 ng/mL), recombinant Human IL-18 (10 ng/mL), and recombinant Human IL-21 (10 ng/mL) (R&D Systems, USA) for 48 h, and then replaced with activated medium (270 ng/mL recombinant Human IL-2, 20 ng/mL recombinant Human IL-12, 20 ng/mL recombinant Human IL-18, and 20 ng/mL recombinant Human IL-21) for further culture. Cell density was maintained at 1 × 10^6^ cells/mL. On day 28 of culture, a small number of autologous NK cells were collected for quality control and intravenously reinjected along with autologous NK cells at a density of 4.15 × 10^9^ (3.76–5.87 × 10^9^) cells, with a infusion time of 30 min. The experimental process was carried out in accordance with the Good Clinical Practice. The time interval for the second blood collection was about 37 (25–57) days.

### Isolation of human peripheral blood T cells and NK cells

Fresh blood was obtained from healthy volunteers after informed consent was provided, and the protocol was approved by the institutional review board of China Pharmaceutical University (Permit Number: SYXK2016-0011). Lymphoprep™ (STEMCELL Technologies, Canada) was used to isolate human PBMCs. Human CD3^+^ T cells were obtained from PBMCs using human CD3 T-cell sorting magnetic beads (Miltenyi Biotec, Germany) in accordance with the manufacturer’s protocol. NK cells were isolated from peripheral blood using the RosetteSep™ Human NK Cell Enrichment Cocktail (STEMCELL Technologies) according to the manufacturer’s protocol.

### Separation and expansion of mouse NK cells in vitro

Mouse splenic NK cells were isolated using NK Cell Isolation Kit (Miltenyi Biotec) according to the manufacturer’s instructions. Briefly, mouse spleens were obtained, spleen cells were filtered using a 70 µm cell filter, and spleen lymphocytes were obtained by density gradient centrifugation using a mouse spleen lymphocyte separation kit (Solarbio, China). The splenic lymphocytes were collected to obtain high-purity NK cells using magnetic beads to separate mouse NK cells. The isolated splenic NK cells were cultured in maintenance RPMI-1640 medium supplemented with 10% foetal bovine serum (FBS), 1% L-glutamine, 1% penicillin/streptomycin, 1% minimal essential medium (MEM) non-essential amino acid, 1% sodium pyruvate acid, and 50 µM mercaptoethanol (all from Life Technologies, USA). In addition, 200 IU/mL recombinant interleukin 2 (rIL-2) (PeproTech, USA) and 10 ng/mL mouse IL-15 (mIL-15) (PeproTech) were added to the medium. Then 10^6^ NK cells and 10^6^ irradiated splenic lymphocytes were collected, and 5 mL amplification medium (maintenance medium supplemented with 1000 IU/mL rIL-2, 10 ng/mL mIL-15, and 30 ng/mL anti-NKp46 antibody (PA5-46986, Invitrogen, USA)) was added to a T25 flask. Every three days, 1000 IU/mL rIL-2 was added and fresh amplification medium was added on the 9th day to maintain the cell density at 1 × 10^6^ cells/mL. NK cells were collected on the 20th day, and NK cells were amplified 50-fold. Then the purity of the NK cells was determined by flow cytometry.

### Extraction of preadipocytes and muscle satellite cells

The preadipocytes were extracted as previously described [11]. Briefly, human or mouse fat was removed under sterile conditions and cut into 1–2 mm of pieces, washed twice with PBS, and digested with collagenase II (Solarbio) at 37 °C for 1 h. Then cells were filtered with a 100 μm cell filter, centrifuged at 300 g for 5 min, and collected. The adherent cells were cultured in α-MEM supplemented with 20% FBS for 12 h, and digested with trypsin to collect adherent cells. The muscle satellite cells were isolated as previously described [34]. The quadriceps muscle was cut into 1-2 mm of pieces, and 5 mL collagenase II was added for digestion at 37 °C for 12 min. The mixture was mixed with a pipette, and 5 mL complete medium was added after additional digestion for 12 min. The cells were filtered using a 70 μm cell filter and centrifuged at 300 g for 5 min. Then cells were cultured with 5 mL medium added daily for 4 days. The adherent cells were blown away with a pipette and centrifuged at 2000 × g for 5 min. After trypsin digestion at 37 °C for 5 min, cells were centrifuged at 2000 × g for 5 min and collected. Then 5 mL F-10 Ham’s medium supplemented with 20% FBS and 4 ng/mL basic fibroblast growth factor (PeproTech) was added.

### Cytotoxicity of mouse splenic NK cells to SNCs was detected by the lactate dehydrogenase assay

The SNCs were induced by 0.2 μM Adriamycin (MedChemExpress, USA) for 24 h or by continued culture for 20 days after 10 Gy X-ray radiation. Then 5000 SNCs were placed into a plate, and splenic NK cells (cell viability: 93.5–97.1%) were added at effector to target ratios of 50:1, 25:1, 12.5:1, 6.25:1, 3.125:1, and 1.563:1. The supernatants were collected after co-culturing at 37 °C for 24 h, and a lactate dehydrogenase (LDH) cytotoxicity detection kit (Cayman Chemical, USA) was used for detection. Cytotoxicity was calculated by the following formula: cytotoxicity (%) = (mixture cell experiment − target cell spontaneous − effector cell spontaneous)/(target cell maximum − target cell spontaneous) × 100.

### Live imaging

Twelve 10-week-old C57BL /6 mice were purchased from Changzhou Cavens Laboratory Animal Co., Ltd. (Jiangsu, China). Then 1 × 10^6^ 1,1′-dioctadecyl-3,3,3′,3′-tetramethylindocarbocyanine iodide (DiR) (Invitrogen)-labeled control preadipocytes or radiation-induced senescent preadipocytes were resuspended in 200 µL phosphate-buffered saline (PBS) and injected into the abdominal cavity of mice with a 22 gauge needle. After three days, 5 × 10^6^ allogenic NK cells (cell viability: 93.6–96.2%) in 100 µL PBS were injected through the tail vein. On the 10th day, the mice were anesthetized with isoflurane. Fluorescence was detected using the IVIS 100 Series In Vivo Imaging System (PerkinElmer, MA, USA).

### Flow cytometry

To detect the apoptosis of SNCs co-incubated with DiR-labeled splenic NK cells (cell viability: 91.8–93.2%), 1 × 10^5^ SNCs were digested with accutase at 37 °C for 10 min and then stained with an Annexin V and Propidium Iodide (PI) Apoptosis Staining Kit (Multisciences Biotech Co., Ltd., China) according to the manufacturer’s protocol. SNCs were gated in the DiR-negative position. To test the activation of NK cells, mouse NK cells were collected and stained with mNK1.1-allophycocyanin (APC) (S17016D) and with mouse cluster of differentiation 69-R-phycoerythrin-cyanine7 (mCD69-PE-Cy7) (H1.2F3) (Biolegend, USA) at 4 °C for 30 min. Then cells were processed with the Cytodex/Cytoperm Plus Kit (BD Biosciences, USA) and stained with mouse interferon gamma-brilliant violet 421 (mIFNγ-BV421) (XMG1.2) at 4 °C for 30 min. Perforin-APC (S16009A) staining of human NK cells was performed using the same protocol as that used for extracellular staining (CD56-fluorescein isothiocyanate [FITC] (5.1H11), CD69-PE (FN50)) and intracellular staining (perforin-APC) (Biolegend).

To detect peripheral blood NK cells, 100 µL anticoagulant blood was collected. For mouse peripheral blood, CD3-FITC, NK1.1-APC, and CD69-PE-Cy7 were added. For human peripheral blood, CD3-BV421 (OKT3) and CD56-FITC were added and incubated for 20 min at room temperature. Then 400 µL 1× erythrocyte lysate (BD Biosciences) was added and incubated for 3 min, followed by three washes with PBS. To detect cell proliferation in adipose tissue, the mice were intraperitoneally injected with 200 µL of 10 mg/mL bromodeoxyuridine (BrdU) at 24 and 72 h before euthanasia. After euthanasia, the inguinal fat depots were removed and cut into fragments, digested at 37 °C for 30 min with collagenase II and DNase, and filtered using a 100 µm cell filter. Cells were processed with the Cytofix/Cytoperm Plus Kit and stained with BrdU-FITC (3D4) and Ki67-PE (16A8). Flow cytometry was performed on an CytoFLEX flow cytometer. The LIVE/DEAD Fixable Violet Dead Cell Stain Kit (Thermo Fisher Scientific, USA) was used to exclude dead cells in all experiments. Data were analysed using FlowJo software (FlowJo LLC, Ashland, OR, USA).

### Reverse transcription-quantitative PCR

RNA was extracted using Trizol reagent and reversed transcribed into cDNA using Hifair® II 1st Strand cDNA Synthesis Kit (Yeasen Biotechnology, China). Quantitative PCR (qPCR) was performed using the reaction mix of SYBR Green (Yeasen Biotechnology) on the ABI QuantStudio 3 Real-Time PCR System (Applied Biosystems, USA), with GAPDH serving as an internal control. Data were analysed using the 2−ΔΔCt method. The primer sequences list is reported in Supplementary Material (Table 2–3).

### Senescence-associated β-galactosidase staining

For cell staining, cells were washed once with PBS, fixed in senescence-associated β-galactosidase (SA-*β*-gal) solution (Cell Signaling Technology, USA) at room temperature for 15 min, washed three times with PBS, and incubated overnight in SA-*β*-gal staining solution at 37 °C. The plate was sealed with parafilm to prevent evaporation of the staining medium. Cells were washed with PBS and observed with a microscope. For frozen section staining, the sections were dried at 37 °C for 30 min and fixed at room temperature in SA-*β*-gal fixation solution for 15 min. The sections were washed three times with PBS and incubated overnight in SA-*β*-gal staining solution at 37 °C. Then they were stained with eosin for 1 min and rinsed with water for 2 min. SA-*β*-gal^+^ staining data were analysed using Image-Pro Plus 6.0 software.

### Human adipose tissue explants

Adipose tissue from three individuals was obtained by liposuction. One of the subjects was a male, and two were females. The mean age of the subjects was 57.0 ± 7.6 years (mean ± S.D.; range, 50–65). Mean BMI was 40.5 ± 5.1 kg/m^2^ (mean ± S.D.; range, 36.7–46.2). The ethics committee of Shanghai Mengchao Cancer Hospital (No.05, 2020, Medical Ethics Committee of Shanghai Mengchao Cancer Hospital) approved the experimental scheme. Informed consent was obtained for all volunteers. Adipose tissue was cut into small pieces with a diameter of about 2 mm. Five pieces of tissue were placed in each well of a 96-wells plate, and 200 µL of either preadipocytes or conditioned medium from radiation-induced senescent preadipocytes, supplemented with 10% human AB serum, 1% L-glutamine, 1% penicillin/streptomycin, 1% MEM non-essential amino acids, and 1% sodium pyruvate acid, were added to the co-culture for 24 h. Then the medium was replaced with fresh medium containing 5 ×10^5^ autologous NK cells (cell viability: 92.3–95.7%) and cultured for another 48 h, after which NK cells were collected for flow cytometry. The adipose tissue was washed five times with PBS, and the same medium was added for a further culture for 48 h; the supernatant (100 µL) was used for multi-factor detection. Preadipocytes or radiation-induced senescent preadipocytes were derived from the adipose tissue of the same individual.

### Western blotting

Total protein’s content was obtained by lysing the adipose tissue with RIPA lysis buffer containing PMSF; the protein concentration was determined using a BCA protein quantification kit. Proteins were resolved by 12% sodium dodecyl sulfate-polyacrylamide gel electrophoresis and then electrotransferred to nitrocellulose membranes. Membranes were blocked in 5% skim milk for 2 h at room temperature and incubated at 4 °C overnight with the following antibodies: P16 (F-12, 1:1000; Santa Cruz, USA), P21 (EPR3993, 1:1500; Abcam, USA), CREB (WL01848, 1:1500; Wanleibio, China), p-CREB (AF5785, 1:1000; Beyotime Biotechnology, China), and GAPDH (RK-200-301-A33, 1:1000; Multisciences Biotech Co., Ltd.). Membranes were incubated with the secondary antibody (70-GAM0072, 1:20000) for 1 h at room temperature, and protein’s content were detected using an enhanced chemiluminescent kit.

### Content of cyclic adenosine monophosphate in NK cells was detected by enzyme-linked immunosorbent assay

Splenic NK cells (3 × 10^5^) (cell viability: 95.1–96.7%) were collected after co-incubation and resuspended with 300 µL PBS containing 10 µM 3-Isobutyl-1-methylxanthine (Beyotime Biotechnology). Cells were broken using an ultrasound crusher and were centrifuged at 3000 g for 15 min at 4 °C. The supernatants were measured for cyclic adenosine monophosphate (cAMP) levels using the cAMP ELISA Kit (Elabscience, Bethesda, USA) according to the manufacturer’s instructions.

### Multiplex protein analyses

Conditioned medium (50 μL) was collected, and the levels of proinflammatory cytokines and chemokines were detected on the Luminex X-200 System using the Human XL Cytokine Luminex Premixed Kit according to the manufacturer’s protocol (R&D Systems, USA).

### Enzyme-linked immunosorbent assay analysis of DA in peripheral blood

Twenty-two 18-month-old C57BL/6 mice were purchased from Hangzhou Ziyuan Experimental Laboratory Animal Technology Co., Ltd. (Hangzhou, China). Acein was injected intraperitoneally at 5, 10, and 20 mg/kg. The mice were anesthetized with isoflurane at 10 min and 1, 2, and 4 h after Acein administration followed by retro-orbital blood collection (80 µL) and centrifugation at 1000 g for 15 min to obtain plasma. Plasma DA content was measured using a DA ELISA Kit (Elabscience, USA) according to the manufacturer’s instructions.

### Cytometric bead array assay

Mouse blood was centrifuged at 1000 g for 15 min to obtain plasma. Plasma levels of IL-6, tumour necrosis factor alpha (TNF-α), and IL-1α were measured using the CBA Mouse IL-6 Flex Set, CBA Mouse TNF Flex Set, and ALP CBA Mouse IL-1 Flex Set (BD Pharmingen) according to the manufacturer’s instructions. Human blood was centrifuged at1000 g for 15 min to obtain plasma. Plasma levels of IL-6, TNF-α, and IL-10 were measured using the CBA Human IL-6 Flex Set, CBA Human TNF Flex Set, and CBA Human IL-10 Flex Set (BD Pharmingen) according to the manufacturer’s instructions. Data were analysed using FCAP Array v3 software.

### Analysis of serum biochemical indexes

Blood was collected, kept at room temperature for 2 h, and centrifuged at 1000 g for 10 min to obtain serum. The serum levels of alanine aminotransaminase (ALT), aspartate transaminase (AST), creatinine (CREA), blood urea nitrogen (BUN), uric acid (UA), and total cholesterol (CHOl) were detected using the Roche P800 automatic biochemical analyzer (Roche Diagnostics Co., Indianapolis, IN, USA).

### Detection of tissue NAD

Liver tissue or adipose tissue (30 mg) was homogenized with NAD^+^/NADH extract solution, and the supernatant was collected by centrifugation at 1000 g for 10 min. The NAD^+^/NADH Quantification Kit (Beyotime Biotechnology) was used for detection according to the manufacturer’s instructions.

### Mice and treatment

The Committee for Animal Use of China Pharmaceutical University (Permit Number: SYXK2016-0011) approved all animal experiments. Twenty-five 18-month-old female C57BL/6 mice were purchased from Hangzhou Ziyuan Experimental Laboratory Animal Technology Co., Ltd. (Hangzhou, China), and raised in a specific pathogen-free environment in a 12:12 h light-dark cycle. All animals were provided food and water ad libitum. Acein (H-Pro-Pro-Thr-Thr-Thr-Lys-Phe-Ala-Ala-OH) was synthesized by GL Biochem Ltd (China) (Supplementary Fig. 6A, B). After mice rose to 20-month-old, they were randomly divided into four groups: PBS, Acein, NK cell, and NK cell + Acein. PBS was used to adjust the concentration of NK cells (cell viability: 91.7–95.3%) to 5 × 10^6^ cells/ mL, and 100 µL NK cells were reinfused through the tail vein twice a month. 2 h after NK cell infusion, 10 mg/kg Acein was injected intraperitoneally twice a day, for consecutive 3 days. The dosing regimen lasted 4 months.

### Statistical analyses

For data analyses, the P value was analysed with default parameters in GraphPad Prism 8 by the unpaired nonparametric *t*-test (comparison between two groups) and one-way analysis of variance (ANOVA) (comparison of more than two groups). Data are expressed as the mean ± standard deviation (SD); n represents the number of experiments and the number of animals. *P* less than 0.05 was considered statistically significant.

## Discussion

This study assessed the ability of Acein and NK cell combination therapy to remove the accumulation of SNCs in tissues. We found that adoptive infusion of about 10^9^ NK cells in humans effectively eliminated SA-β-gal-positive CD3^+^ T cells in peripheral blood, and decreased the expression of age-related genes in T cells. Importantly, the cytotoxic effects of NK cells on SNCs have also been demonstrated on human adipose tissue, in which tissue senescence markers and senescence-related secretion phenotypes are decreased. In vitro, NK cells exerted cytotoxicity against SNCs from different sources, and DA could enhance the killing activity of NK cells against SNCs. In aged mice, NK cells combined with Acein that induces DA-secretion in the brain, significantly reduced senescence markers and SASP in multiple tissues. These results demonstrate the potential of this combination therapy in eliminating SNCs.

In most cases, the initiation of senescence is induced by the DNA damage response (DDR) [35], which often triggers the innate and adaptive immune processes [36]. DDR is critical in inducing SNCs to up regulate NKG2D ligands, which enables NK cells to recognize and clear SNCs in a timely manner [37]. In our study, the activation ligands of NK cells were up regulated and the expression of inhibitory ligands was down regulated in both radiation-induced and doxorubicin-induced SNCs. In addition, the activation of NK cells by SNCs was also manifested by the increased expression levels of CD69 and IFNγ in NK cells, which indicated that indeed NK cells had the function of immune monitoring of SNCs.

Adoptive NK cell therapy has been evaluated in clinical trials for the treatment of melanoma, renal cell carcinoma, lymphoma, and breast cancer. The infused cells were able to expand in the body but did not cause a tumour response [38]. The main reasons might be that the infiltration of NK cells into solid tumours is limited and the inhibitory microenvironment of tumours weakens the activity of NK cells [39]. In contrast, normal tissues do not have the dense structures of solid tumours that significantly prevent NK cell infiltration. In addition, the SASP of SNCs will produce a large number of chemokines and will maintain the local inflammatory environment [40], which in turn is conducive for NK cell migration to SNCs and the production of cytotoxic effects against SNCs. It is worth noting that in clinical trials of adoptive NK cell infusion to treat tumours, patients have already undergone a large amount of pre-treatment, which may also affect the efficacy of NK cells [41]. The NK cells used for senescence clearance were from healthy donors and were significantly more energetic. In this study, NK cells from young mice were selected as effector cells to evaluate the therapeutic effects of the combined therapy in the aged mouse, considering that NK cells from young mice might have a better ability to clear SNCs. Corresponding with possible treatment in humans, cell freezing technology can be adopted to preserve NK cells in a timely manner at a young age [42]. We can also continue to explore the possibility of infusing allogeneic NK cells such as umbilical cord blood- and stem cell-derived NK cells [43].

As a highly complex system of the body, the immune system and nerve cells must interact with each other to function normally [44]. These two systems can be regulated by soluble factors, among which DA is one of the key transmitters [21]. DA acts by interacting with DRs. Previous studies have shown that the DR is expressed on NK cells [45]. Treatment of mouse spleen NK cells with SKF-38393 (D1-like DR agonist) enhances NK cytotoxicity [24], while the D2-like DR agonist quinpirole attenuates cytotoxicity [46]. In this study, the addition of DA significantly increased the cytotoxicity of NK cells against SNCs, and the level of cAMP and phosphorylation level of CREB in NK cells increased. The D1-like DR mediated this synergistic effect. Although these might be the reasons why DA enhances NK cells to clear SNCs, the specific mechanism remains to be explored.

DA hydrochloride is only suitable for some extreme indications such as myocardial infarction, trauma, endotoxin sepsis, cardiac surgery, renal failure, congestive heart failure and other shock syndrome. Accordingly, the treatment of stimulating endogenous DA secretion might become a mild DA supplementation regimen. Acein interacts with angiotensin converting enzyme with high affinity stimulating the release of DA from the brain [26]. In this study, we demonstrated that intraperitoneal injection of Acein also increased the level of DA in peripheral blood. NK cells combined with Acein effectively reduced the aging phenotype in the aging mouse, and the efficacy was better than that of NK cells alone. This study is only a preliminary attempt to show the intervention of aging using adoptive infusion of NK cells combined with Acein. The favourable efficacy of this treatment will encourage us to continue to explore the detailed mechanism of the synergistic effect of Acein on NK cells as well as the optimal dose of Acein combined with NK cells for a maximal biological effect. In addition, future studies are needed to determine the concentration difference of Acein-induced peripheral DA release in different tissues and the optimal DA concentration required for the better clearance of NK cells against SNCs in different tissues.

## Conclusion

The results of this study showed that NK cells could produce significant cytotoxicity against SNCs, and DA had significant synergistic effects on NK cells to clear SNCs. In the aging mouse model, adoptive NK cell infusion combined with Acein significantly reduced the expression of tissue senescence markers and age-related genes. These results support further exploration of this combined therapy.

## Supplementary information

Supplementary materials

Reproducibility checklist

## Data Availability

All data supporting the findings of this study are available within the article and the supplementary materials.
