# Supplementary material for: Combining adoptive NK cell infusion with a dopamine-releasing peptide reduces senescent cells in aged mice
Source: Cell Death Dis. 2022 Apr 5;13(4):305. doi: 10.1038/s41419-022-04562-w (PMC8983684; doi:10.1038/s41419-022-04562-w)
Supplement: Supplementary file 1 — Supplementary materials [file 41419_2022_4562_MOESM1_ESM.docx]

**Supplementary materials**

**Combining adoptive NK cell infusion with a dopamine-releasing peptide reduces senescent cells in aged mice**

**Zongke Bai^1,2^**^#^**, Peiwei Yang^1,2^**^#^**, Fan Yu^1,2^, Zhong Li^3^, Zheng Yao^1,2^, Jean Martinez^4^, Mengwei Li^1,2^, Hanmei Xu^1,2^***

1 The Engineering Research Center of Synthetic Polypeptide Drug Discovery and Evaluation, Jiangsu Province, China Pharmaceutical University, Nanjing 210009, P.R. China.

2 State Key Laboratory of Natural Medicines, Ministry of Education, China Pharmaceutical

University, Nanjing 210009, P.R. China.

3 Shanghai Engineering Research Center for Cell Therapy, Shanghai 201805, China

4 Faculté de Pharmacie, Institut des Biomolécules Max Mousseron (IBMM) UMR 5247, Université de Montpellier, CNRS, ENSCM, Montpellier, France.

# These authors contributed equally to this work.

* Corresponding Author: Hanmei Xu, Tel: 86-25-83271007; Fax: 86-25-83271007; E-mail: [13913925346@126.com](mailto:13913925346@126.com); Address: 639 Longmian Avenue, Jiangning District, Nanjing City, Jiangsu Province, China


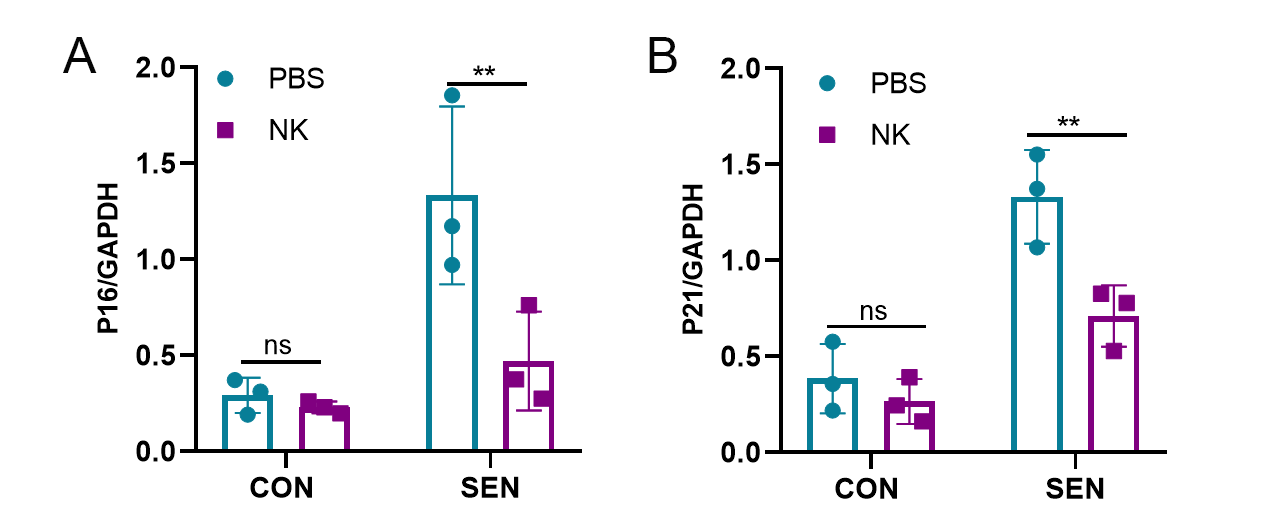


**Figure 1: Senescent markers for P16 and P21 were detected by Western blotting.** A. The densitometry quantification of the protein levels of P16 against GAPDH. B. The densitometry quantification of the protein levels of P21 against GAPDH. n=3. Data are presented as means ± SD. Differences were assessed by the one-way ANOVA test. ns P﹥0.05, ** P < 0.01.


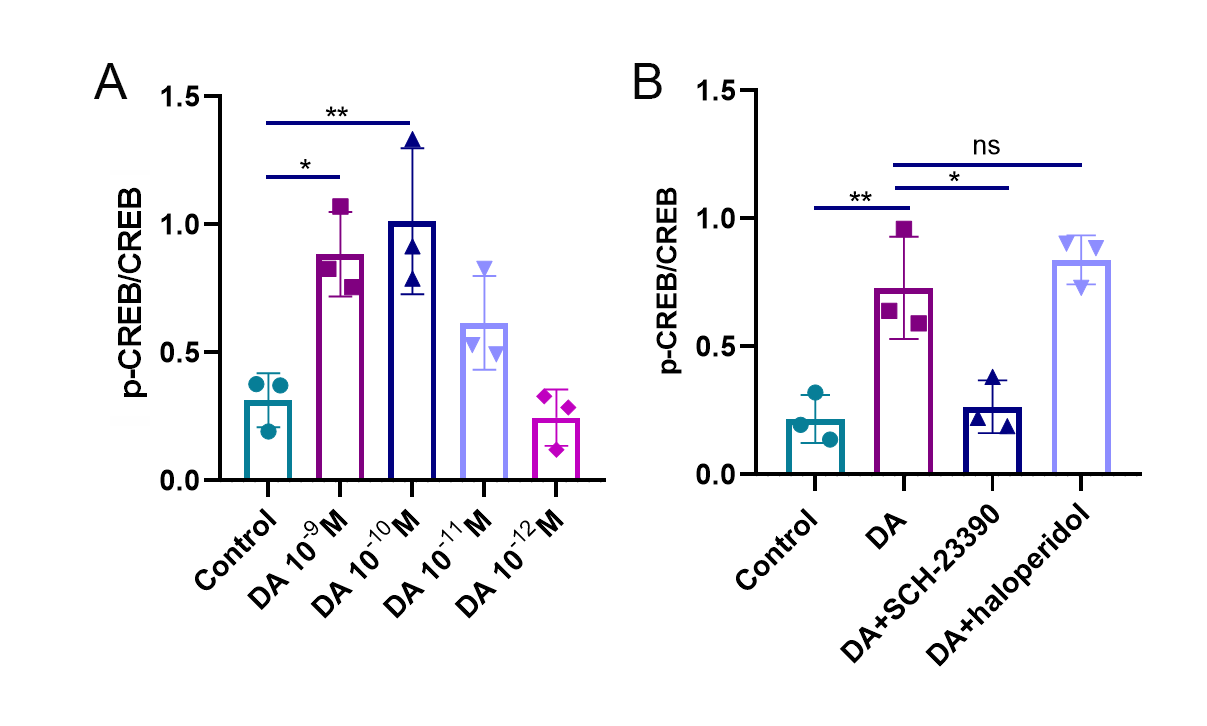


**Figure 2: Phosphor-CREB in NK cells was detected by Western blotting.** A-B. The densitometry quantification of the protein levels of phosphor-CREB against CREB. n=3. Data are presented as means ± SD. Differences were assessed by the one-way ANOVA test. ns P﹥0.05, * P < 0.05, ** P < 0.01.


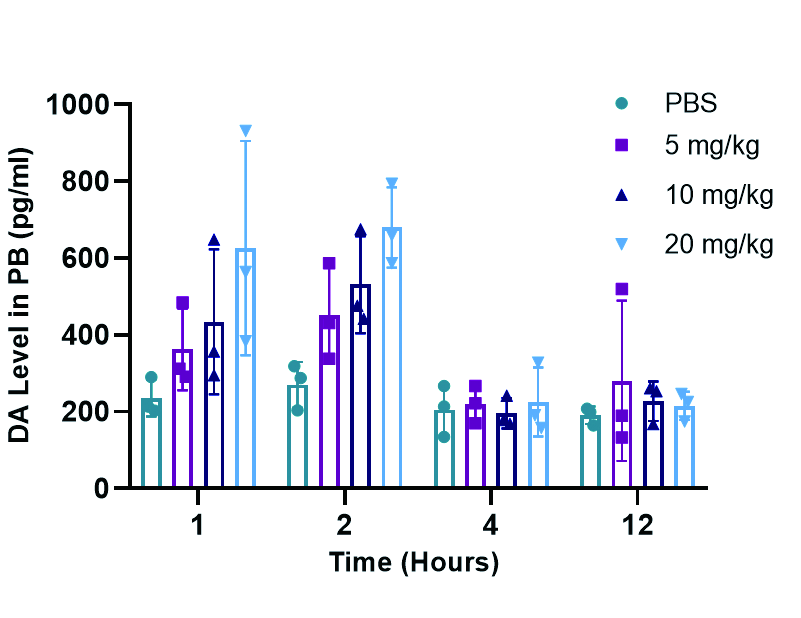


**Figure 3: Induction of peripheral dopamine release by Acein.** Peripheral dopamine levels induced by several concentrations of Acein at different time point in old mice. n=3. Data are presented as means ± SD. Differences were assessed by the two-way ANOVA test. ns P﹥0.05.


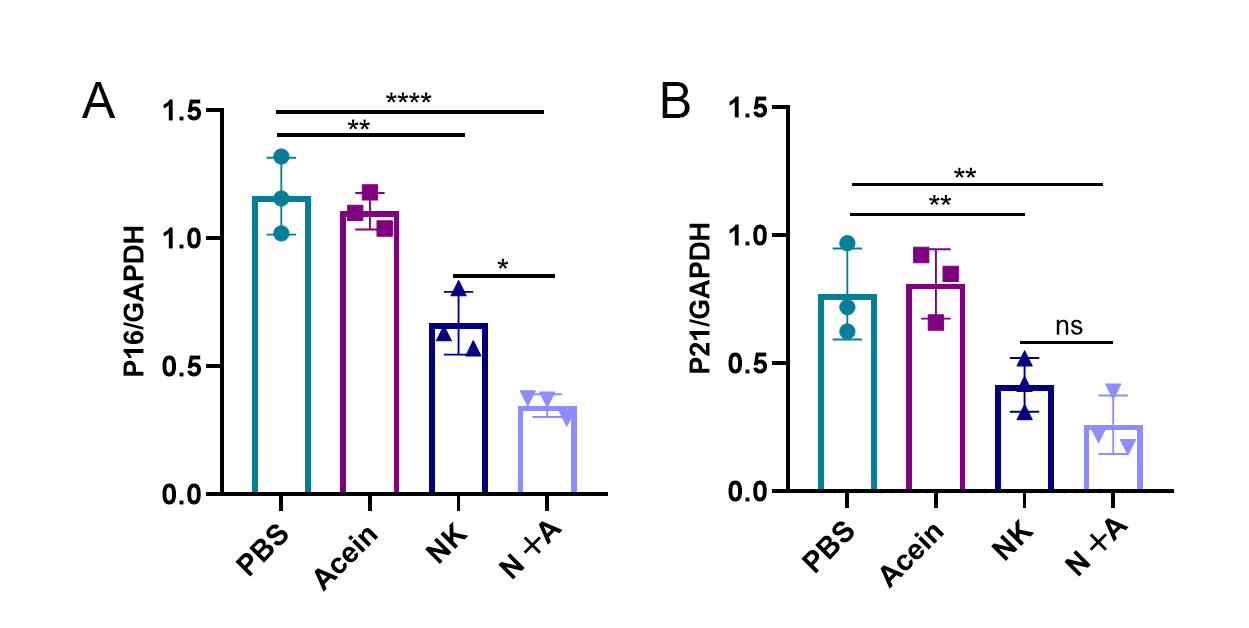


**Figure 4: Senescent markers for P16 and P21 were detected by Western blotting.** A. The densitometry quantification of the protein levels of P16 against GAPDH. B. The densitometry quantification of the protein levels of P21 against GAPDH. n=3. Data are presented as means ± SD. Differences were assessed by the one-way ANOVA test. ns P﹥0.05, * P < 0.05, ** P < 0.01, **** P < 0.0001.


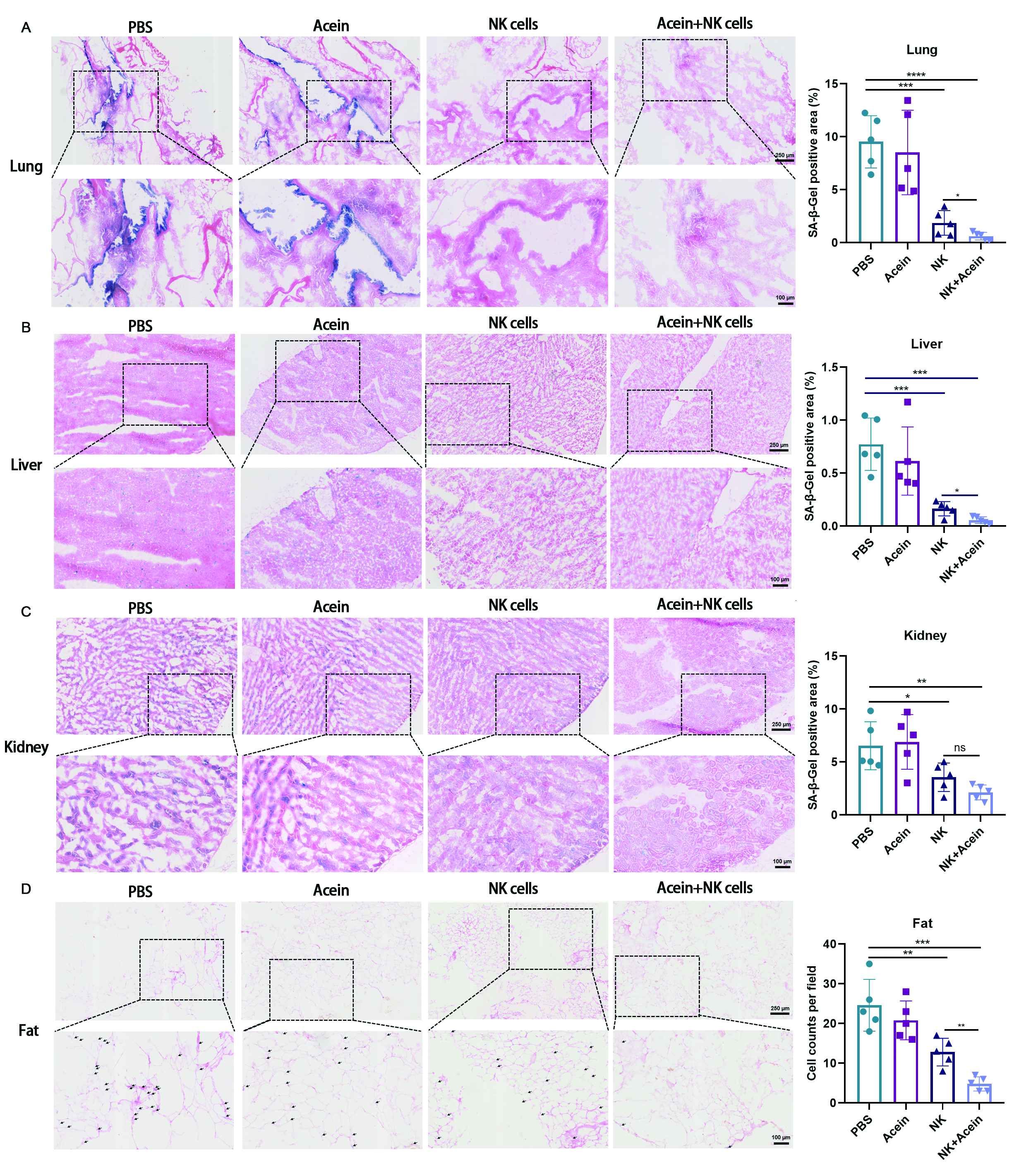


**Figure 5:** **NK cells combined with acein eliminate SNCs in different tissues in aged mouse.** Representative images (left) and quantiﬁcation (right) of SA-β-gal staining in the lungs **(A).** livers **(B).** kidneys **(C).** and fat **(D).** from aged mice after treatments. n=5. Data are presented as means ± SD. Differences were assessed by the one-way ANOVA test. ns P﹥0.05, * P < 0.05, ** P < 0.01, *** P < 0.001, **** P < 0.0001.


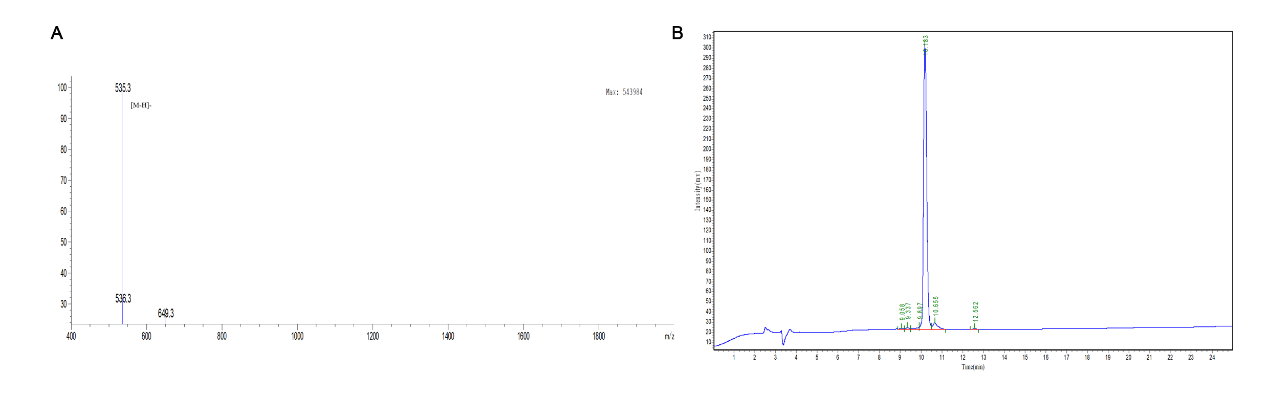


**Figure 6: Characterization of acein. A.** Characteristic ion peaks of acein from mass spectrometry. **B.** The purity of acein was identified by HPLC.

**Table 1:** **Biochemical Indexes of 26 individuals having received NK cells administration**

| Biochemical Indexes | Pre-infusion | After-infusion | P Value |
| --- | --- | --- | --- |
| ALT (U/L) | 25.069±12.532 | 23.308±7.786 | 0.5763 |
| AST (U/L) | 22.019±6.794 | 21.003±8.532 | 0.6880 |
| UA (µmol/L) | 320.644±128.318 | 306.808±91.208 | 0.1350 |
| BUN (mmol/L) | 4.079±1.232 | 4.138±1.549 | 0.0962 |
| CREA (µmol/L) | 69.938±13.309 | 69.885±13.556 | 0.9906 |

**Table 2: Primers for speciﬁc genes of mouse**

| Gene | Forward Primer (5 ′ to 3 ′) | Reverse Primer (5 ′ to 3 ′) |
| --- | --- | --- |
| *p16* | CGCAGGTTCTTGGTCACTGT | TGTTCACGAAAGCCAGAGCG |
| *p21* | CCTGGTGATGTCCGACCTG | CCATGAGCGCATCGCAATC |
| *il6* | GTTCTCTGGGAAATCGTGGA | GGTACTCCAGAAGACCAGAGGA |
| *mcp1* | TTAAAAACCTGGATCGGAACCAA | GCATTAGCTTCAGATTTACGGGT |
| *pai1* | GCATGCCTGACATGTTTAGTG | GTTTACCTCGATCCTGACCTTT |
| *H60b* | AGCCTGAGAGAGCTTTCAGAA | GGGTGTCAGAATTATGTTGGGAG |
| *Ulbp1* | CTGCCAGTAACAAGGTCCTTTC | GCTGTTCCTATGAGCACCAATG |
| *Raet1e* | TGACCAAGCGCCATCATTTTAT | TTCACGTCACACCAGGGAAGG |
| *Tap-1* | GGACTTGCCTTGTTCCGAGAG | GCTGCCACATAACTGATAGCGA |
| *B2m* | TTCTGGTGCTTGTCTCACTGA | CAGTATGTTCGGCTTCCCATTC |
| *H2-K1* | CAGGTGGAGCCCGAGTATTG | CGTACATCCGTTGGAACGTG |
| *D1dr* | TGTGACACGAGGTTGAGC | GGTGGTCTGGCAGTTCTT |
| *D2dr* | CCATTGTCTG GGTCCTGT | TGCCCTTGAG TGGTGTCT |
| *D3dr* | CTACGCCCTG TCCTACTGT | CCACCTGTCA CCTCCAAG |
| *D4dr* | GTGTTGGACG CCTTTCTTCG | GGGTTGAGGG CACTGTTGA |
| *D5dr* | CTGCGAGCAT CCATCAAG | CACAAGGGAA GCCAGTCC |
| *Gapdh* | CTTTGTCAAGCTCATTTCCTGG | TCTTGCTCAGTGTCCTTGC |

**Table 3: Primers for speciﬁc genes of human**

| Gene | Forward Primer (5 ′ to 3 ′) | Reverse Primer (5 ′ to 3 ′) |
| --- | --- | --- |
| *p16* | CCCGATTGAAAGAACCAGAGAG | AGTTGTGGCCCTGTAGGA |
| *p21* | TGTCCGTCAGAACCCATGC | AAAGTCGAAGTTCCATCGCTC |
| *il6* | ACTCACCTCTTCAGAACGAATTG | CCATCTTTGGAAGGTTCAGGTTG |
| *mcp1* | TCATAGCAGCCACCTTCATTC | CTCTGCACTGAGATCTTCCTATTG |
| *pai1* | AGTGGACTTTTCAGAGGTGGA | GCCGTTGAAGTAGAGGGCATT |
| *Gapdh* | CTGGGCTACACTGAGCACC | AAGTGGTCGTTGAGGGCAATG |
